# Overexpression of the GR Riborepressor LncRNA GAS5 Results in Poor Treatment Response and Early Relapse in Childhood B-ALL

**DOI:** 10.3390/cancers13236064

**Published:** 2021-12-01

**Authors:** Marieta Xagorari, Antonios Marmarinos, Lydia Kossiva, Margarita Baka, Dimitrios Doganis, Marina Servitzoglou, Maria Tsolia, Andreas Scorilas, Margaritis Avgeris, Dimitrios Gourgiotis

**Affiliations:** 1 Laboratory of Clinical Biochemistry—Molecular Diagnostics, Second Department of Pediatrics, School of Medicine, National and Kapodistrian University of Athens, “P. & A. Kyriakou” Children’s Hospital, 11527 Athens, Greece; mxagor@med.uoa.gr (M.X.); antmar@med.uoa.gr (A.M.); 2 Second Department of Pediatrics, School of Medicine, National and Kapodistrian University of Athens, “P. & A. Kyriakou” Children’s Hospital, 11527 Athens, Greece; lydiak@med.uoa.gr (L.K.); mtsolia@med.uoa.gr (M.T.); 3 Department of Pediatric Oncology, “P. & A. Kyriakou” Children’s Hospital, 11527 Athens, Greece; margbaka@hotmail.com (M.B.); doganisd@gmail.com (D.D.); mservitzoglou@gmail.com (M.S.); 4 Department of Biochemistry and Molecular Biology, Faculty of Biology, National and Kapodistrian University of Athens, 15771 Athens, Greece; ascorilas@biol.uoa.gr

**Keywords:** growth arrest-specific 5, non-coding RNA, long non-coding RNA, leukemia, pediatric leukemia, childhood leukemia, glucocorticoid receptor, glucocorticoids, BFM, ALL IC-BFM

## Abstract

**Simple Summary:**

Although childhood acute lymphoblastic leukemia (chALL) management is considered as one of the success stories in modern clinical oncology, the increased incidence of relapse in high-risk patients and the severe toxicity/long-term health effects due to chemotherapy intensity highlight the need for further improvements in patients’ risk stratification and personalized management. Synthetic glucocorticoids (GCs) are the core agents in chALL chemotherapy, exerting their role through the nuclear glucocorticoid receptor (GR), while GAS5 lncRNA suppresses the GCs-GR axis through binding to GR’s DNA binding domain. The objective of the study was the evaluation of GAS5 prognostic utility in chALL. GAS5 overexpression was strongly associated with a higher risk for short-term relapse and poor treatment outcome, independently of patients’ clinicopathological data. Moreover, “GAS5-including” multivariate models resulted in superior risk stratification and clinical benefit for disease prognosis, supporting precision medicine decisions in chALL.

**Abstract:**

Glucocorticoids (GCs) remain the cornerstone of childhood acute lymphoblastic leukemia (chALL) therapy, exerting their cytotoxic effects through binding and activating of the glucocorticoid receptor (GR). GAS5 lncRNA acts as a potent riborepressor of GR transcriptional activity, and thus targeting GAS5 in GC-treated chALL could provide further insights into GC resistance and support personalized treatment decisions. Herein, to study the clinical utility of GAS5 in chALL prognosis and chemotherapy response, GAS5 expression was quantified by RT-qPCR in bone marrow samples of chB-ALL patients at diagnosis (*n* = 164) and at end-of-induction (*n* = 109), treated with ALL-BFM protocol. Patients’ relapse and death were used as clinical end-points for survival analysis. Bootstrap analysis was performed for internal validation, and decision curve analysis assessed the clinical net benefit for chALL prognosis. Our findings demonstrated the elevated GAS5 levels in blasts of chALL patients compared to controls and the significantly higher risk for short-term relapse and poor treatment outcome of patients overexpressing GAS5, independently of their clinicopathological data. The unfavorable prognostic value of GAS5 overexpression was strongly validated in the high-risk/stem-cell transplantation subgroup. Finally, multivariate models incorporating GAS5 levels resulted in superior risk stratification and clinical benefit for chALL prognostication, supporting personalized prognosis and precision medicine decisions in chALL.

## 1. Introduction

Childhood acute lymphoblastic leukemia (chALL) is the most common type of cancer in children, accounting for ~26% of all childhood malignancies up to the age of 15 years worldwide [1,2]. B-cell ALL (B-ALL) represents 75–80% of overall childhood leukemia cases. During the past decades, there has been significant progress in disease management, resulting in >90% of 5-year survival rates of chALL patients, mostly due to risk-adapted chemotherapy, use of novel therapeutic agents, understanding of the disease molecular genetics, and the use of hematopoietic stem-cell transplantation (HSCT) [3,4].

The current treatment of chALL relies on a multidrug chemotherapy regimen delivered in sequential phases (induction, consolidation, reinduction, and maintenance) over a period of 2 to 3 years [5,6,7]. Based on their strong anti-inflammatory and immune-suppressive functions, two synthetic glucocorticoids (GCs), prednisone/prednisolone and dexamethasone, have been widely used in the treatment of hematological malignancies [8,9,10]. GCs exert their cytotoxic effects through binding and activating the glucocorticoid receptor (GR), a ligand-dependent transcription factor of the family of nuclear steroid receptors. Following binding of GCs in the cytoplasm, GRs are homodimerized, translocate to the nucleus, and interact with glucocorticoid response elements (GRE) to promote gene expression (transactivation pathway), or they remain monomeric and repress the activity of transcription factors AP-1 or NF-κB (transrepression pathway) [11,12]. GCs-GR signaling results in inhibition of inflammation, as well as induction of cell cycle arrest, growth arrest, and apoptosis, to exert their therapeutic role [13].

The evaluation of sensitivity to GCs, performed by the number of peripheral leukemic blasts on day 8 of induction therapy (prednisone response), remains a major prognostic factor and criteria for risk classification and chemotherapy adjustment [14,15]. Poor prednisone response (≥1000 blasts/μL) is strongly associated with increased risk for disease relapse and worse prognosis. In addition, secondary resistance to GC therapy is highly prevalent in relapsed ALL and is responsible for therapeutic failure and patient death. Several recent studies have been focused on addressing the basis of GC resistance, mostly by directly targeting *NR3C1* inactivating genetic aberrations, as well as GR expression and post-translational modifications [16,17,18]. In this regard, altered expression of GR isoforms, most importantly the increased expression of the transcriptionally inactive GRβ nuclear isoform, loss-of-function mutations and polymorphisms of *NR3C1*, and phosphorylation of specific serine residues of GR have been associated with attenuated GR signaling and GC resistance. However, the biological and molecular background of GC resistance in chALL is only partially disclosed and used in clinical settings, highlighting the need for further clinical and translational research in GC-treated leukemias.

Long non-coding RNAs (lncRNAs) are spliced, 5′-capped, and 3′-polyadenylated ssRNAs >200 nt, without open reading frame (ORF) and protein-coding ability. Along with miRNAs, lncRNAs represent the best-studied classes of ncRNAs, emerging as the most powerful regulators of gene expression in epigenetic and post-transcriptional levels [19,20]. The growth arrest-specific 5 (GAS5) lncRNA, originally identified to be accumulated in cDNA libraries from growth-arrested cells [21], is encoded by the intergenic *GAS5* on 1q25.1 and represents one of the most highly transcribed ncRNAs in the human genome [22]. Despite the presence of a 5-terminal oligopyrimidine tract sequence (5′-TOP) and a short ORF (for ~50 amino acids), the *GAS5* is poorly conserved and currently does not encode for a functional polypeptide [23,24]. GAS5 has been actively implicated in numerous physiological procedures, including growth/cell cycle arrest and induction of apoptosis [25]. Similarly to most 5′-TOP RNAs, GAS5 levels are controlled (degraded) by nonsense-mediated decay in normally dividing cells, while upon exposure to cellular stressors, such as serum deprivation, density arrest, or drugs, translation is inhibited resulting in GAS5 accumulation toward cell growth/proliferation arrest favoring survival [23,26,27,28]. Mechanistically, GAS5 has been demonstrated to act as a potent riborepressor of GR as well as of other steroid hormone receptors (SRs) with an affinity for GREs. To exert this role, the 3′-terminal of GAS5 (nucleotides 546–566) forms a putative stem-loop structure mimicking GREs that interacts directly with GR/SRs DNA binding domain (DBD) and represses their transcriptional activity [29,30]. In this regard, GAS5 was demonstrated to be necessary for normal growth arrest and mTOR antagonists-mediated inhibition of proliferation both in normal and leukemic T cells [31,32,33].

Despite the well-documented role in GCs-GR signaling suppression and regulation of cell growth, the clinical utility of GAS5 in chALL has not been completely evaluated yet. Herein, we have studied, for the first time, the prognostic value of GAS5 lncRNA in improving risk stratification and prediction of chemotherapy response in chB-ALL patients treated according to ALL-BFM protocols.

## 2. Materials and Methods

### 2.1. Study Patients’ Cohort

Bone marrow (BM) specimens were obtained by aspiration from 164 children with newly diagnosed chB-ALL at diagnosis. Patients were treated according to the BFM backbone protocol in the “P. & A. Kyriakou” Children’s Hospital, Athens, Greece. Additionally, matched BM specimens at the end-of-induction (EoI; day 33) were included (based on sample availability) from 109 of the patients. Patients’ stratification was performed according to ALL-BFM 95 and ALL-IC BFM 2009 guidelines. Clinicopathological data, including the evaluation of MRD on days 15 and 33, prednisone response on day 8, and of BM blasts percentage (BM response) on days 15 and 33, were recorded for all patients. Finally, 70 BM specimens from children not suffering from any hematological or other types of malignancy were included as normal controls.

Informed consent was obtained from all parents and legal guardians of patients prior to participation in the study. The study was approved by the ethics committee of “P. & A. Kyriakou” Children’ s Hospital (Ref: 15527, on 13 November 2015), and was conducted according to the standards of the 1975 Declaration of Helsinki, as revised in 2008.

### 2.2. Cytogenetics

For cytogenetic analysis, unstimulated BM cells were directly prepared by 24 h culture in RPMI 1540 culture medium with 25% fetal calf serum at 37 °C. The International System for Cytogenetic Nomenclature (ISCN) was used for the classification of metaphase chromosomes following analysis by standard G-banding technique.

### 2.3. Immunophenotype Analysis

Immunohistotype analysis of BM aspirates was performed via multiparametric flow cytometry (MFC) with an FC-500 cytometer (Beckman Coulter Inc., Nyon, Switzerland) using CXP software. Blasts were analyzed using a panel of monoclonal antibodies against the lymphoid differentiation antigens: HLA-DR+, cTdt, cIgM, CD10, CD11a, CD19, CD20, CD22, CD24, CD34, CD38, CD58, and cCD79a. The presence of a surface and/or an intracellular antigen was confirmed when at least 20% and 10% of the cells expressed it, respectively. Lineage assignment was conducted based on EGIL recommendations. External quality analysis was performed via the NEQAS program.

### 2.4. MRD Evaluation

Minimal residual disease (MRD) was assessed at days 15 and 33 by MFC analysis (see Section 2.3), using a lineage-specific panel: CD9, CD10, CD11a, CD19, CD20, CD22, CD34, CD38, CD45, and CD58 according to BFM protocol guidelines. A cut-off level of 0.01% is set for the interpretation of an MRD result as positive.

### 2.5. Isolation of Total RNA

Total RNA was isolated from BM specimens with TRI-Reagent BD (Molecular Research Center, Inc., Cincinnati, OH, USA) according to the manufacturer’s protocol, dissolved in RNA Storage Solution (Ambion, Austin, TX, USA), and stored at −80 °C until analysis. Total RNA concentration and purity were evaluated spectrophotometrically at 260 and 280 nm using NanoDrop 1000 Spectrophotometer (Thermo Fisher Scientific, Waltham, MA, USA), while RNA integrity was visually confirmed by agarose gel electrophoresis.

### 2.6. First-Strand cDNA Synthesis

First-strand cDNA synthesis of poly(A) RNAs occurred in a 20 μL reaction containing 50 U MMLV reverse transcriptase (Invitrogen, Carlsbad, CA, USA), 40 U RNaseOUT recombinant ribonuclease inhibitor (Invitrogen), 5 μM oligo-dT primers, and 1 μg total RNA template. Reverse transcription was performed at 37 °C for 60 min, while MMLV inactivation occurred at 70 °C for 15 min.

### 2.7. Quantitative Real-Time PCR (qPCR)

GAS5 levels were quantified by SYBR Green-based qPCR. Specific primers for GAS5 (F: 5′-CTTGCCTGGACCAGCTTAAT-3′, R: 5′-CAAGCCGACTCTCCATACCT-3′) and *GAPDH* (F: 5′-ATGGGGAAGGTGAAGGTCG-3′, R: 5′-GGGTCATTGATGGCAACAATATC-3′) were designed based on published sequences (NCBI Ref Seq: NR_002578.3 for GAS5 and NR_152150.2 for *GAPDH*) and in silico specificity analysis, for the amplification of a 122 bp GAS5-specific and a 107 bp *GAPDH*-specific amplicons.

The 7500 Real-Time PCR System (Applied Biosystems, Carlsbad, CA, USA) was used for the qPCR analysis. The 10 μL reactions consisted of KAPA SYBR^®^ Fast Universal 2× qPCR MasterMix (Kapa Biosystems, Inc., Woburn, MA, USA), 200 nM of each specific PCR primer, and 10 ng of cDNA template. The thermal protocol consisted of a polymerase activation step at 95 °C for 3 min, followed by 40 cycles of denaturation at 95 °C for 15 s, and primer annealing and extension at 60 °C for 1 min. Following amplification, melting curve analysis and agarose gel electrophoresis were performed to detect specific amplicons from non-specific products and/or primer dimers.

For each target, technical duplicate reactions were performed, and the average Ct was used in the quantification analysis. No template controls were included in each run. The quantification of GAS5 levels was conducted by the 2^−ΔΔCT^ relative quantification method using *GAPDH* as the endogenous reference gene for data normalization.

### 2.8. Statistical Analysis

IBM SPSS Statistics 20 (IBM Corp., Armonk, New York, NY, USA) was used for the statistical analysis. Sapiro-Wilk and Kolmogorov-Smirnov tests were applied to test the normal distribution of the data. GAS5 expression differences between chALL patients and controls or between diagnosis and EoI of chALL patients were assessed by Mann-Whitney *U* and Wilcoxon signed-rank tests, respectively. The ability of GAS5 to discriminate chALL patients and controls was evaluated by ROC curve and logistic regression analyses. The non-parametric Mann-Whitney *U* and Kruskal-Wallis tests were used appropriately to study the correlation of GAS5 levels with patients’ clinicopathological data.

Survival analysis was performed by Kaplan-Meier curves and Cox proportional regression analysis. Post-treatment disease relapse and patients’ death were used as clinical end-point events, while the X-tile algorithm was used for the adoption of an optimal cut-off value of GAS5 levels. Internal validation was performed by bootstrap analysis based on 1000 bootstrap samples. Decision curve analysis (DCA), according to Vickers et al. [34], was applied by STATA 16 software (StataCorp LLC., College Station, TX, USA) toward the evaluation of GAS5 clinical benefit in chALL prognosis and treatment response.

## 3. Results

### 3.1. Baseline Clinical and Experimental Data

**Figure 1:**
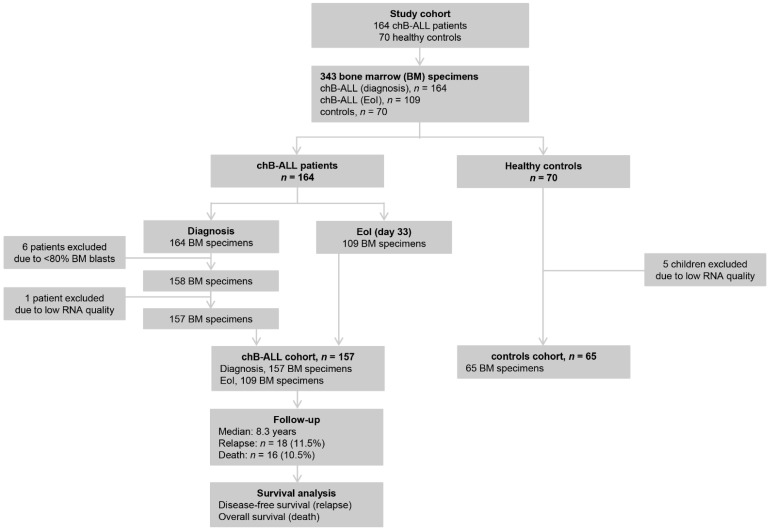
REMARK diagram of the study.

The flow diagram of the study is included in Figure 1. Of the 164 patient-derived BM samples, 6 specimens were excluded from the analysis due to <80% BM blast percentage, and 1 sample was excluded due to low RNA quality. The patients’ cohort consisted of 157 children suffering from precursor B-ALL (96.2% CD10+ and 3.8% CD10−) with median age of 5 years old and median % BM blasts percentage of 91.0% (Table 1). Most of the patients were males (56.7%) with <20,000 WBC/μL at diagnosis (65.6%). Cytogenetic analysis revealed the presence of ETV6-RUNX1 translocation and high hyperdiploidy (>50 chromosomes) in 26.9% and 17.3% of the patients, respectively. Regarding the response to treatment induction, poor prednisone response (≥1000 blasts/mL) at day 8 and M2-M3 BM response (≥5% blasts) at day 15 were observed in 12 (7.6%) and 28 patients (17.8%), respectively, while 4 patients (2.5%) were characterized with M2 BM response at EoI (day 33). According to available MRD data on day 15 (129 patients) and on day 33 (114 patients), 96 (74.4%) and 21 patients (18.4%) were found positive, respectively. Based on BFM guidelines, 110 (70.1%) and 27 (17.2%) patients were stratified as intermediate- and high-/very-high-risk, respectively. Patients were followed-up for a median period of 99 months (~8.3 years; reverse Kaplan–Meier method), in which 18 patients relapsed (11.5%) and 16 patients died (10.2%), while the mean disease-free survival (DFS) and overall survival (OS) intervals were 158.1 months (95% CI: 149.7–167.4) and 161.4 months (95% CI: 153.3–169.6), respectively. Finally, of the 70 healthy children-derived BM samples, 5 specimens were not included in the study due to low RNA quality. Most of the controls were males (50.8%) with a median age of 5 years old, while no significant heterogeneity in age (*p* = 0.191) or sex (*p* = 0.374) was revealed between patients and controls.

### 3.2. GAS5 Is Overexpressed in chALL Patients and Decreases to Normal Levels at the EoI

**Figure 2:**
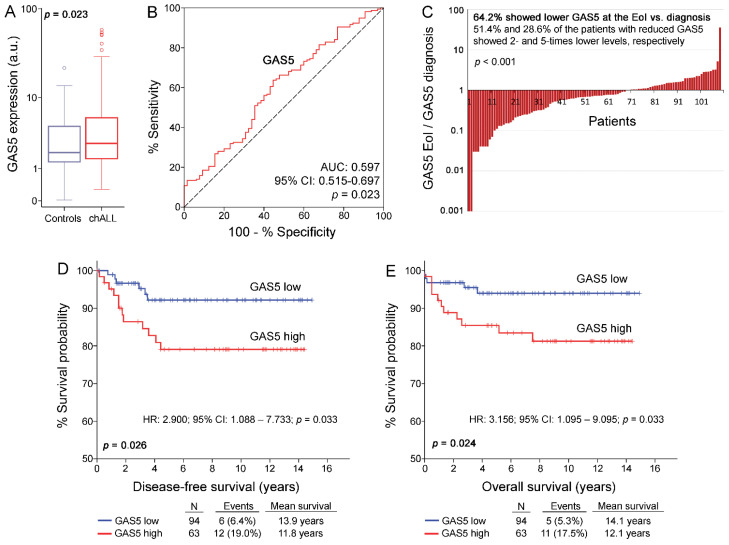
GAS5 is overexpressed in blasts of chB-ALL patients and correlates with higher risk short-term relapse and poor post-treatment survival. (**A**) Box plot presenting GAS5 levels in BM specimens of chALL patients at diagnosis compared to healthy children (controls). *p*-value calculated by Mann-Whitney *U* test. (**B**) ROC curve analysis of GAS5 levels for the discrimination of chALL patients from healthy controls. AUC: area under the curve, 95% CI: 95% confidence intervals. *p*-value calculated by the Hanley and McNeil method. (**C**) Bar graph of the ratio of GAS5 levels at the EoI compared to diagnosis. *p*-value calculated by Wilcoxon Signed-Rank test. (**D**,**E**) Kaplan-Meier curves for the DFS (**D**) and OS (**E**) of chALL patients according to GAS5 levels at diagnosis. *p*-values were calculated by log-rank test.

The descriptive statistics of GAS5 levels in BM specimens of chALL (diagnosis and EoI) and healthy controls are included in Supplementary Table S1. The analysis highlighted that GAS5 is overexpressed in chALL patients at disease diagnosis compared to healthy children (*p* = 0.023; Figure 2A). Moreover, ROC analysis (AUC: 0.597; 95% CI: 0.515–0.697; *p* = 0.023; Figure 2B) and univariate logistic regression (OR: 2.399; 95% CI: 1.253–4.594; *p* = 0.008; Supplementary Table S2) validated the increased GAS5 levels in chALL and highlighted their value for the discrimination of chALL patients from healthy controls. Interestingly, significantly reduced GAS5 levels were highlighted at the EoI treatment (day 33) compared to disease diagnosis (*p* < 0.001; Figure 2C). More precisely, of the 109 enrolled patients with matched BM specimens at the EoI, 70 (64.2%) patients showed lower GAS5 expression at the EoI vs. 39 (35.8%) patients with increased GAS5 levels compared to diagnosis. Finally, the degree of GAS5 downregulation is highlighted by the fact that approximately 51.4% and 28.6% of the patients with reduced GAS5 expression at the EoI showed 2- and 5-times lower GAS5 levels, respectively. The analysis of GAS5 expression with established prognostic markers of chALL, including patients’ age, cytogenetics, WBC, prednisone response at day 8, BM response at days 15 and 33, and BFM risk groups, did not highlight any statistically significant association.

### 3.3. chALL Patients Overexpressing GAS5 Are at Significantly Higher Risk for Short-Term Relapse and Poor Treatment Outcome

chALL patients were stratified, treated, and monitored according to the BFM backbone protocol. Disease relapse and patients’ death are used as clinical end-point events for the DFS and OS analysis. No significant differences in survival outcomes (OS and DFS) as well as in clinicopathological and treatment response data were revealed between patients treated with ALL-BFM 95 and ALL IC-BFM 2009 protocols. Using the X-tile algorithm, the 60th percentile of blasts GAS5 levels at disease diagnosis was adopted as the optimal cut-off value for the classification of chALL patients to “GAS5-high” and “GAS5-low” groups.

**Figure 3:**
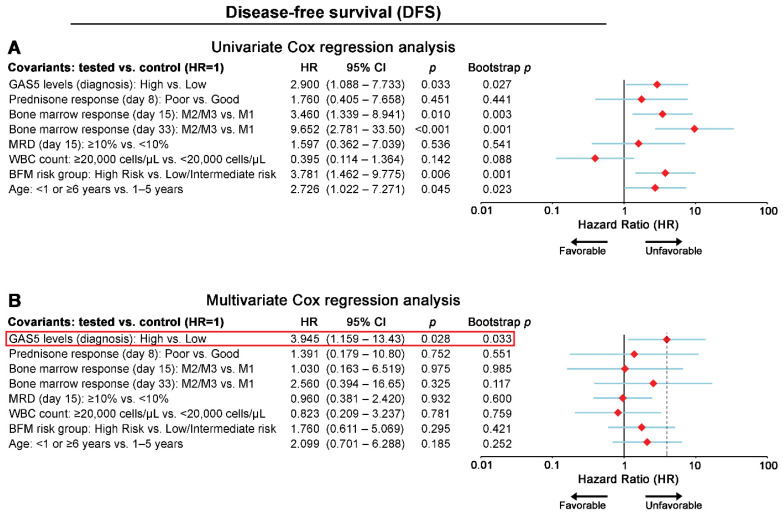
GAS5 overexpression at diagnosis represents an independent predictor of chALL patients’ short-term relapse. Forest plots of the univariate (**A**) and multivariate (**B**) Cox regression analysis for patients’ DFS. Internal validation was performed by bootstrap Cox proportional regression analysis based on 1000 bootstrap samples. HR: hazard ratio; 95% CI: 95% confidence interval of the estimated HR intervals.

Kaplan-Meier survival curves highlighted the significantly worse DFS (*p* = 0.026; Figure 2D) and OS (*p* = 0.024; Figure 2E) outcome of the chALL patients with elevated GAS5 blasts levels compared to those with low/moderate levels. Univariate Cox (Figure 3 and Supplementary Table S3) regression analysis confirmed that “GAS5-high” chALL patients at disease diagnosis are at significant higher risk for poor treatment outcome, as highlighted by the increased risk for short-term relapse (HR: 2.900; 95% CI: 1.088–7.733; *p* = 0.033) or patients’ death (HR: 3.156; 95% CI: 1.095–9.095; *p* = 0.033).

**Figure 4:**
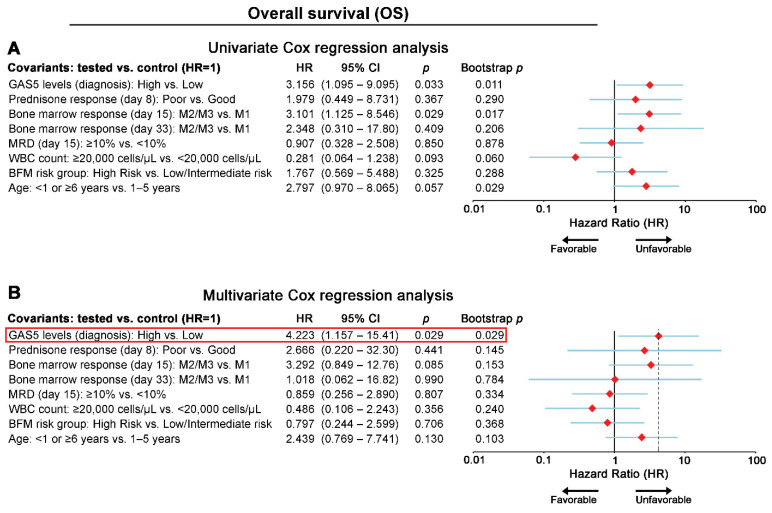
GAS5 overexpression at diagnosis represents an independent predictor of chALL patients’ worse survival. Forest plots of the univariate (**A**) and multivariate (**B**) Cox regression analysis for patients’ OS. Internal validation was performed by bootstrap Cox proportional regression analysis based on 1000 bootstrap samples. HR: hazard ratio; 95% CI: 95% confidence interval of the estimated HR intervals.

To evaluate the independent clinical value of GAS5 expression in the chALL treatment course, multivariate Cox regression models (Figure 3 and Figure 4) were adjusted against the established and clinically used prognostic markers of chALL, while bootstrap analysis was used for internal validation. In this regard, multivariate models were adjusted for GAS5 expression, WBC, prednisone response at day 8, BM response at days 15 and 33, MRD at day 15, patients’ risk group, and age. The multivariate models clearly highlighted the independent prognostic strength of blasts GAS5 overexpression at chALL diagnosis for the short-term post-treatment relapse (HR: 3.945; 95% CI: 1.159–13.43; *p* = 0.028; Figure 3) and death (HR: 4.223; 95% CI: 1.157–15.41; *p* = 0.029; Figure 4) of the patients.

### 3.4. GAS5 Significantly Benefits the Prognostic Value of chALL Clinically Used Markers

The independent clinical value of GAS5 overexpression for chALL treatment and survival outcome prompted us to evaluate GAS5’s utility in improving the prognostic strength of the main clinically used chALL markers. Based on ALL IC-BFM 2009 guidelines, WBC ≥ 20,000 cells/μL, poor prednisone response at day 8, M2-M3 responses at day 15, and MRD ≥ 0.1% at day 15 represent the established disease markers for poor treatment response and survival outcome of the patients.

**Figure 5:**
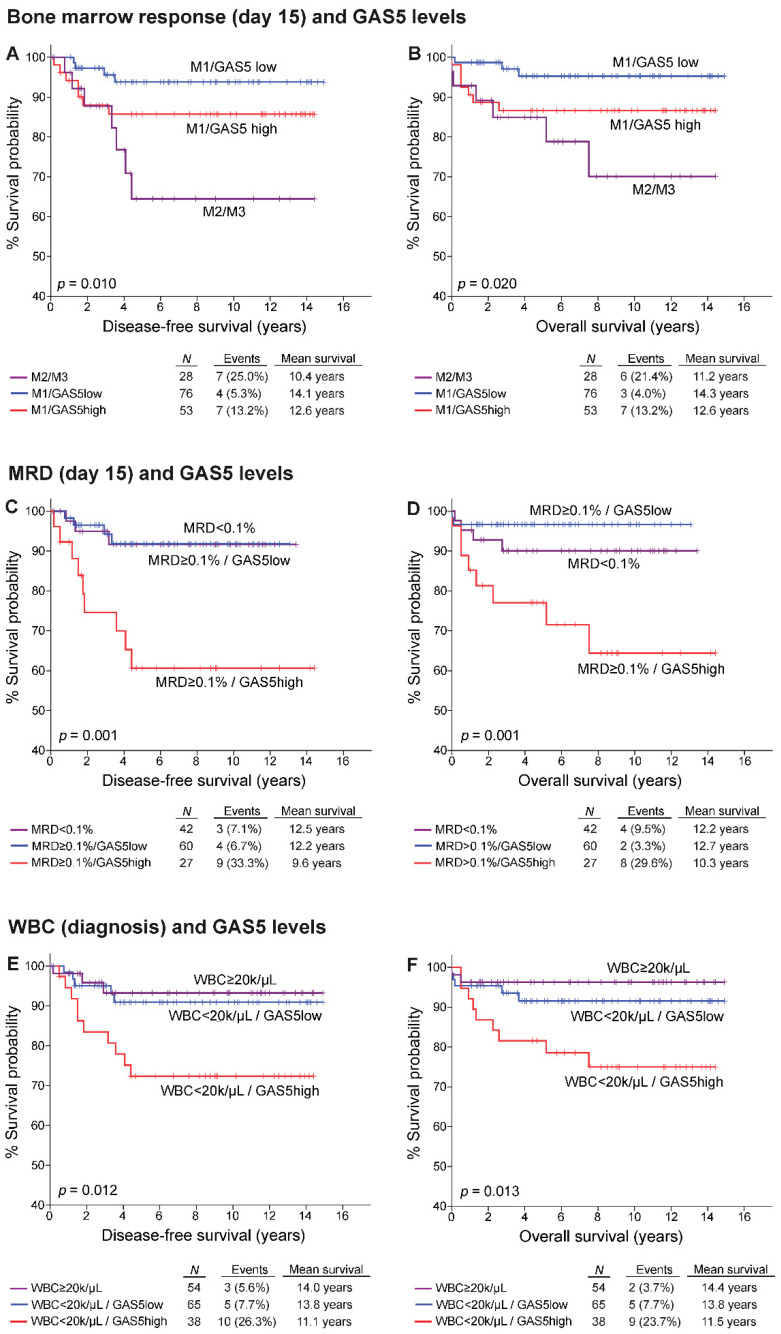
GAS5 levels evaluation improves significantly chALL patients’ risk stratification according to the clinically established prognostic markers. Kaplan-Meier survival curves of the patients DFS and OS according to the combination of GAS5 levels at diagnosis with BM response (day 15; (**A**,**B**)), MRD (day 15; (**C**,**D**)) and WBC at disease (**E**,**F**). *p*-values were calculated by log-rank test.

Evaluation of GAS5 levels with the above-mentioned markers resulted in significantly improved risk stratification and prognostic specificity (Figure 5). More precisely, “GAS5-high” chALL patients at disease diagnosis were demonstrated to suffer from significantly higher risk for short-term relapse and poor survival expectancy, despite the presence of favorable disease markers, such as WBC < 20,000 cells/μL (*p* = 0.012 for DFS; *p* = 0.013 for OS) or M1 response at day 15 (*p* = 0.010 for DFS; *p* = 0.020 for OS). Moreover, MRD at day 15 of the induction protocol represents the most reliable and efficient marker for risk stratification and adjustment of treatment intensity. In this regard, evaluation of GAS5 expression ameliorated significantly the predictive strength of MRD at day 15, as MRD ≥ 0.1% patients overexpressing GAS5 presented significantly worse DFS (*p* < 0.001) and OS (*p* = 0.001) compared to MRD ≥ 0.1% ”GAS5-low” patients, who revealed to resemble MRD < 0.1% group treatment outcome.

### 3.5. DCA Demonstrated the Clinical Benefit of GAS5 Evaluation in chALL Prognosis

**Figure 6:**
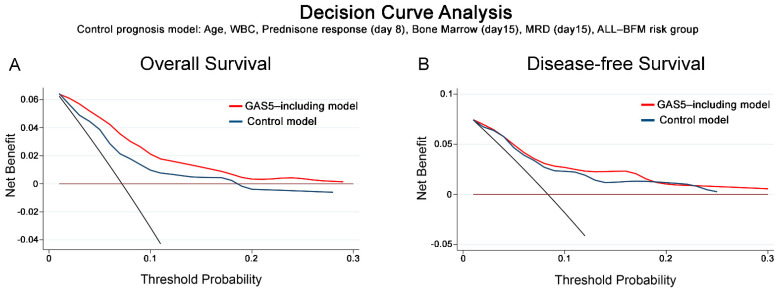
Decision curve analysis (DCA) highlights the superior clinical benefit of multivariate prognostic models incorporating GAS5 expression. DCA curves of “control” and “GAS5-including” multivariate prognostic models for patients’ OS (**A**) and DFS (**B**). Net benefit is plotted against various ranges of threshold probabilities.

DCA, according to Vickers et al. [34], was used for the evaluation of the clinical benefit of multivariate prognostic models integrating GAS5 expression with the established clinical markers of chALL prognosis (Figure 6). DCA curves clearly demonstrated the superior clinical net benefit of multivariate models incorporating blasts’ GAS5 overexpression at disease diagnosis for the prognosis of both OS (Figure 5A) and DFS (Figure 5B) post-treatment outcome, compared to the “control” models of the established clinical markers of patients’ age, WBC, prednisone response (day 8), bone marrow (day15), MRD (day15) and ALL-BFM risk group, from low threshold probabilities <10%.

### 3.6. High-Risk Patients Overexpressing GAS5 Demonstrated Poor Treatment Response

Risk stratification of chALL patients was performed according to ALL IC-BFM 2009 guidelines. In this regard, 27 (17.2%) patients were classified as high-risk, with 12 (44.4%) of them having undergone stem-cell transplantation. According to ALL-BFM guidelines, high-risk treatment involves an intensified consolidation phase using GCs administration, in contrast to standard- and intermediate-risk patients’ GC-free consolidation phase. In this regard, we hypothesized that blast GAS5 levels could predict the treatment response and the survival outcome of the high-risk group.

**Figure 7:**
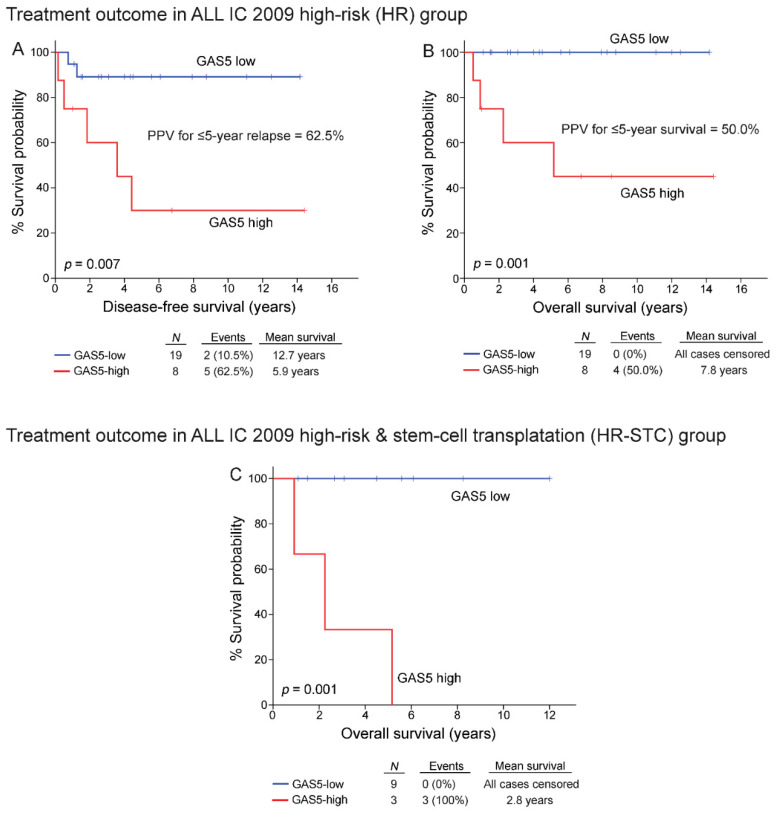
High-risk (HR) patients overexpressing GAS5 are at a significantly greater risk for poor response to BFM treatment. Kaplan-Meier survival curves for the DFS (**A**) and OS (**B**) of the HR group, as well as the OS of the HR group patients that underwent stem-cell transplantation (**C**) according to GAS5 levels at diagnosis. *p*-values calculated by log-rank test.

Indeed, the survival analysis highlighted the poor treatment outcome of the high-risk patients overexpressing GAS5 compared to the “GAS5-low” group, as demonstrated by the stronger risk for short-term relapse (*p* = 0.007; median DFS: 3.6 years; Figure 7A) and death (*p* = 0.001; median OS: 5.2 years; Figure 7B). In this regard, the positive prediction value (PPV) of GAS5 overexpression for ≤5-year DFS and ≤5-year OS of the high-risk chALL patients were 62.5% and 50%, respectively. Similarly, a significantly increased risk for early post-treatment death (*p* = 0.001; Figure 7C) was confirmed for the “GAS5-high” patients that underwent stem-cell transplantation. To this end, multivariate COX regression models confirmed the greater risk for the high-risk chALL patients overexpressing GAS5 at disease diagnosis for short-term relapse.

## 4. Discussion

Although chALL management is considered as one of the success stories in modern clinical oncology [35], the increased incidence of relapse in high-risk patients along with the severe toxicity/long-term health effects due to chemotherapy intensity highlights the clinical need for further improvements in patients’ risk stratification and personalized management. GCs remain the cornerstone of chALL chemotherapy, while chALL patients’ response/sensitivity to induction treatment GCs (prednisone response) represents a major prognostic and risk-stratification marker in chALL [7,8,9]. The potent anti-inflammatory and immune-suppressive role of GCs is excreted in a ligand-depended manner through GR-mediated modulation of GRE-regulated genes. By a 3′-terminal stem-loop structure that mimics GREs and blocks GR/SR DBD, GAS5 represents a potent riborepressor of GR signaling [29,30], and thus targeting of GAS5 in GC-treated leukemias could provide further insights on GC resistance as well as novel molecular markers to support modern precision medicine.

Despite several recent studies that associate GAS5 with treatment outcome and patients’ survival in multiple cancers [36,37,38], its clinical value for chALL patients has not yet been elucidated. In the present study, we have analyzed GAS5 levels in BM samples of chB-ALL patients and normal controls in order to evaluate GAS5 lncRNA’s clinical impact on chB-ALL prognosis and prediction of treatment response. Increased GAS5 levels were detected in chALL patients at disease diagnosis compared to controls, while most of the patients displayed significantly reduced GAS5 BM levels at the EoI (day 33). In this regard, ROC and logistic regression analysis verified the discriminatory value of GAS5 between chALL patients and healthy (non-ALL) children BM samples. Due to the antiproliferative effects and downregulation in numerous solid malignancies, a widely accepted tumor suppressor role has been attributed to GAS5 [25]. On this topic, the pioneer works of Mourtada-Maarabouni et al. [31,32] have documented that GAS5 is essential for the normal growth arrest and the inhibitory effects of mTOR inhibitors (rapamycin and its analogs) in both leukemic and non-transformed T lymphocytes. Indeed, Frank et al. identified three distinct structural modules of GAS5 that act separately in leukemic T cells, including a 5′-terminal module that inhibits cell cycling and survival, a highly structured core module that regulates mTOR inhibition, and a 3′-terminal stem-loop structure that blocks GR/SR DNA-dependent signaling [33].

At first glance, the documented here elevated GAS5 levels in BM blasts of chALL patients compared to controls, as well as the poor treatment response/survival of the patients overexpressing GAS5 contradicts GAS5 role on cell cycle arrest and apoptosis induction. However, tumor aggressiveness and treatment resistance are strongly correlated with the presence of slow-cycling or mitotically quiescent cancer stem cells (CSCs). Quiescence represents a self-protecting mechanism that prevents “damage” of both normal and CSCs upon microenvironment and/or therapy stress [39,40]. Indeed, CSCs remain quiescent in a steady-state that protects them from conventional chemotherapies, while following therapy discontinuation, they exit quiescence and expand, promoting disease relapse [41,42]. Several recent studies have documented the essential role of GAS5 as a “molecular switch” to control quiescence/stemness in CSCs, including the CD133 + CSCs in pancreatic cancer [43] and HCT116-derived CSCs [44], as well as for self-renewal and pluripotency of mouse embryonic stem cells [45]. Thus, in response to chALL-associated stress of BM microenvironment, due to nutrient deprivation and/or hypoxia, GAS5 overexpression in BM blasts could trigger a CSC-like phenotype that favors their survival and supports our findings on GAS5 downregulation at the EoI (clinical blasts-free BM). Contrary to our findings, Gasic et al. reported higher GAS5 levels at the EoI compared to the diagnosis of chALL patients, however the analysis was performed in different sample type, peripheral blood mononuclear cells (vs. BM blasts in our study) of a rather small cohort, not allowing a safe comparison [46]. Ultimately, future studies incorporating large-scale institutional-independent chALL cohorts will help to clarify the regulation of GAS5 expression in chALL blasts and PBMCs, as well as to validate GAS5 clinical utility on chALL patients’ prognosis and treatment response.

Following therapy, the survival analysis highlighted the significantly higher risk for short-term relapse and the declined survival of the chB-ALL patients with GAS5 overexpression in BM blasts at diagnosis. Interestingly, multivariate Cox regression analysis highlighted the superior and independent prognostic value of GAS5 BM levels compared to the clinically used disease markers. The superior and independent clinical value of GAS5 in chB-ALL was confirmed by the ability of GAS5 overexpression to ameliorate the prognostic accuracy of the established disease markers, including MRD, BM response, and WBC. More precisely, incorporation of GAS5 overexpression with patients’ WBC and BM response could identify children prone to early relapse and poor OS, despite the presence of the favorable M1 response (day15) or <20,000 WBC/μL. In the same manner, evaluation of GAS5 overexpression with MRD (day15) resulted in superior stratification within MRD ≥ 0.1% group, as GAS5-high patients suffered from significantly shorter DFS and poor OS compared to the GAS5-low group, resembling the MRD < 0.1% group. Finally, decision curves verified the clinical benefit for patients’ risk stratification and treatment prognosis of the multivariate prediction models incorporating GAS5 levels with the clinically used disease markers compared to “control” models (excluding GAS5). The superior clinical benefit from low threshold probabilities (<10%) clearly demonstrated the clinical impact of GAS5 evaluation in improving the disease prognostication and supporting more personalized patient management.

The association of blasts’ GAS5 overexpression with poor treatment response and survival is in line with the polymorphic function of GAS5 in the homeostasis of lymphoid cells. Indeed, GAS5 could trigger BM blasts toward a more quiescent phenotype, providing survival advantage and chemotherapy resistance. Additionally, the higher GAS5 levels will antagonize GCs for GR DBD, resulting in repression of GR DNA-dependent signaling and attenuation of GCs therapeutic effect in chALL. This is clearly supported by the poor treatment response of the high-risk chALL patients overexpressing GAS5. Considering the need for intensified therapy of the high-risk/very high-risk chB-ALL patients, ALL-BFM guidelines use GCs administration during the consolidation phase, in contrast to the GCs-free consolidation regimens of the standard- and intermediate-risk groups. Survival analysis demonstrated the early relapse and the worse survival of the GAS5-overexpressing high-risk patients compared to the favorable treatment responses of the GAS5-low group, clearly highlighting a poor therapeutic benefit of GAS5-high patients within the same risk group.

## 5. Conclusions

Considering the role of GAS5 lncRNA as a potent riborepressor of GR transcriptional activity, we have evaluated, for the first time, the prognostic value of GAS5 lncRNA in chALL treatment response and outcome. Our findings highlight the overexpression of GAS5 in BM blasts of chALL patients compared to healthy controls, along with the significantly higher risk for short-term relapse and poor treatment outcome of patients overexpressing GAS5, independently of their clinicopathological data. This was strongly validated in the high-risk/HSCT group, the treatment of which involves a consolidation phase using GCs administration, in contrast to the GC-free consolidation phase of standard- and intermediate-risk patients. Finally, multivariate “GAS5-including” models resulted in superior risk stratification and clinical benefit for chALL prognostication, compared to “control” models of the established and clinically used disease markers, fulfilling the clinical need for personalized treatment decisions in modern chALL precision medicine.

## Figures and Tables

**Table 1:** Clinicopathological features of the childhood ALL patients.

Variables	*N*	%
**Gender**		
Male	89	56.7
Female	68	43.3
**Age**		
1–6 years	82	52.2
6–10 years	26	16.6
<1 or ≥10 years	49	31.2
**WBC**		
<20,000 cells/μL	103	65.6
≥20,000 cells/μL	54	34.4
**High Hyperdiploidy (>50 chrom.)**		
Yes	26	17.3
No	124	82.7
Unknown	7	
**ETV6-RUNX1/t(12;21)(p13;q22)**		
Negative	114	73.1
Positive	42	26.9
Unknown	1	
**Philadelphia chromosome**		
Negative	154	98.1
Positive	3	1.9
**Bone Marrow on day 15**		
M1 (blasts <5%)	129	82.2
M2 (blasts 5–25%)	20	12.7
M3 (blasts ≥25%)	8	5.1
**MRD on day 15**		
<0.1%	42	32.6
≥0.1%–<10%	76	58.9
≥10%	11	8.5
Unknown	28	
**MRD on day 33**		
Negative (<0.01%)	93	81.6
Positive (≥0.01%)	21	18.4
Unknown	43	
**Prednisone response on day 8**		
Good (<1000 blasts)	145	92.4
Poor (≥1000 blasts)	12	7.6
**ALL IC - BFM risk groups**		
Standard Risk	20	12.7
Intermediate Risk	110	70.1
High Risk	27	17.2
**Stem Cell Transplantation**		
No	140	89.2%
Yes	17	10.8%
**Disease relapse**		
Relapse-free	139	88.5
Relapse	18	11.5
**Patients’ survival**		
Alive	141	89.8
Dead	16	10.2

## Data Availability

Data are available upon reasonable request.

## Supplementary Materials

The following are available online at https://www.mdpi.com/article/10.3390/cancers13236064/s1, Supplementary Table S1. Descriptive statistics of GAS5 expression levels in chALL patients (diagnosis and EoI) and healthy cohort; Supplementary Table S2. Logistic regression analysis for the discrimination of chALL from healthy controls bone marrow; Supplementary Table S3. Cox regression analysis for the prediction of chALL patients’ disease-free survival (DFS) and overall survival (OS) according to GAS5 expression.
