# Supplementary material for: Overexpression of the GR Riborepressor LncRNA GAS5 Results in Poor Treatment Response and Early Relapse in Childhood B-ALL
Source: Cancers (Basel). 2021 Dec 1;13(23):6064. doi: 10.3390/cancers13236064 (PMC8656629; doi:10.3390/cancers13236064)
Supplement: Supplementary file 1 [file cancers-13-06064-s001.zip › cancers-1462520-supplementary-final/cancers-1462520_Supplemental Table 1.pdf]

Supplemental Table S1. Descriptive statistics of GAS5 expression levels in chALL patients (diagnosis and EoI) and healthy cohort.

Variables	Mean ± SE	Range	Percentiles				
			10	25	50 (median)	75	90
**ch-ALL (diagnosis) (n=157)**							
GAS5	26.10 ± 9.98	0.34 - 1180.43	1.47	1.90	3.25	7.27	28.69
**healthy controls (n=65)**							
GAS5	4.53 ± 0.64	0.02 - 27.62	1.04	1.66	2.38	5.64	12.94
FC			1.41	1.14	1.37	1.29	2.22
**ch-ALL (diagnosis) (n=125)**							
GAS5	26.10 ± 9.98	0.34 - 1180.43	1.47	1.90	3.25	7.27	28.69
**ch-ALL (EoI; day 33) (n=109)**							
GAS5	4.63 ± 0.65	0.33 – 43.77	1.07	1.58	2.33	4.22	8.80
FC			1.37	1.20	1.39	1.73	3.26

SE: Standard Error
FC: fold change
