# Supplementary material for: Overexpression of the GR Riborepressor LncRNA GAS5 Results in Poor Treatment Response and Early Relapse in Childhood B-ALL
Source: Cancers (Basel). 2021 Dec 1;13(23):6064. doi: 10.3390/cancers13236064 (PMC8656629; doi:10.3390/cancers13236064)
Supplement: Supplementary file 1 [file cancers-13-06064-s001.zip › cancers-1462520-supplementary-final/cancers-1462520_Supplemental Table 2.pdf]

Supplemental Table S2. Logistic regression analysis for the discrimination of chALL from healthy controls bone marrow

	Univariate Analysis			Multivariate Analysis[a]		
**Covariant**	**OR[b]**	**95% CI[c]**	***p*-value[d]**	**OR[b]**	**95% CI[c]**	***p*-value[d]**
**log_{10} GAS5**	2.399	1.253-4.594	0.008	2.341	1.114-4.920	0.025
**Age**	1.070	0.983-1.166	0.119	1.069	0.979-1.167	0.139
**Gender**	1.433	0.733-2.803	0.293	1.229	0.614-2.460	0.560

[a] Multivariate logistic regression models adjusted for log_{10} GAS5, patient's age and gender.
[b] Odds ratio
[c] Confidence interval of the estimated OR
[d] Test for trend
