# Supplementary material for: Overexpression of the GR Riborepressor LncRNA GAS5 Results in Poor Treatment Response and Early Relapse in Childhood B-ALL
Source: Cancers (Basel). 2021 Dec 1;13(23):6064. doi: 10.3390/cancers13236064 (PMC8656629; doi:10.3390/cancers13236064)
Supplement: Supplementary file 1 [file cancers-13-06064-s001.zip › cancers-1462520-supplementary-final/cancers-1462520_Supplementary Table 3.pdf]

Supplemental Table S3. Cox regression analysis for chALL patients' disease-free survival (DFS) and overall survival (OS) according to GAS5 levels.

	Univariate analysis									
	**Disease-free survival (DFS)**					**Overall survival (OS)**				
**Covariant**	**HR[a]**	**95% CI[b]**	***p*-value[c]**	**Bootstrap BCa 95% CI[d]**	**Bootstrap *p*-value[c]**	**HR[a]**	**95% CI[b]**	***p*-value[c]**	**Bootstrap BCa 95% CI[d]**	**Bootstrap *p*-value[c]**
**GAS5 expression**										
GAS5-low	1.00					1.00				
GAS5-high	2.900	1.088-7.733	0.033	1.020-12.92	0.027	3.156	1.095-9.095	0.033	1.198-12.99	0.011
**Prednisone Response**										
GPR (<1000 blasts)	1.00					1.00				
PPR (≥1000 blasts)	1.760	0.405-7.658	0.451	0.044-5.635	0.441	1.979	0.449-8.731	0.367	0.044-6.315	0.290
**Bone marrow (day 15)**										
M1 (<5% blasts)	1.00					1.00				
M2/M3 (≥5% blasts)	3.460	1.339-8.941	0.010	1.104-11.90	0.003	3.101	1.125-8.546	0.029	0.717-9.469	0.017
**Bone marrow (day 33)**										
M1 (<5% blasts)	1.00					1.00				
M2/M3 (≥5% blasts)	9.652	2.781-33.50	0.000	0.049-49.50	0.001	2.348	0.310-17.80	0.409	0.046-12.28	0.206
**MRD (day 15)**										
<10%	1.00					1.00				
≥10%	1.597	0.362-7.039	0.536	0.043-5.033	0.541	0.907	0.328-2.508	0.850	0.204-1.768	0.878
**WBC count**										
<20000 cells/μl	1.00					1.00				
≥20000 cells/μl	0.395	0.114-1.364	0.142	0.026-1.126	0.088	0.281	0.064-1.238	0.093	0.025-0.802	0.060
**BFM risk group**										
Low/Interm. risk	1.00					1.00				
High risk	3.781	1.462-9.775	0.006	1.328-10.06	0.001	1.767	0.569-5.488	0.325	0.383-4.545	0.288
**Age**										
1-5 years	1.00					1.00				
<1 or ≥6 years	2.726	1.022-7.271	0.045	1.071-9.535	0.023	2.797	0.970-8.065	0.057	0.933-15.44	0.029

	Multivariate analysis[e]									
	**Disease-free survival (DFS)**					**Overall survival (OS)**				
**Covariant**	**HR**[a]	**95% CI**[b]	***p*-value**[c]	**Bootstrap BCa 95% CI**[d]	**Bootstrap *p*-value**[c]	**HR**[a]	**95% CI**[b]	***p*-value**[c]	**Bootstrap BCa 95% CI**[d]	**Bootstrap *p*-value**[c]
**GAS5 expression**										
GAS5-low	1.00					1.00				
GAS5-high	3.945	1.159-13.43	0.028	0.930-993.3	0.033	4.223	1.157-15.41	0.029	0.990-69.62	0.029
**Prednisone Response**										
GPR (<1000 blasts)	1.00					1.00				
PPR (≥1000 blasts)	1.391	0.179-10.80	0.752	7.52×10^{-7}-1052	0.551	2.666	0.220-32.30	0.441	2.03×10^{-6}-2708	0.145
**Bone marrow (day 15)**										
M1 (<5% blasts)	1.00					1.00				
M2/M3 (≥5% blasts)	1.030	0.163-6.519	0.975	0.002-21.20	0.985	3.292	0.849-12.76	0.085	0.325-34.02	0.153
**Bone marrow (day 33)**										
M1 (<5% blasts)	1.00					1.00				
M2/M3 (≥5% blasts)	2.560	0.394-16.65	0.325	0.257-9170	0.117	1.018	0.062-16.82	0.990	2.29×10^{-7}-1.53×10^{8}	0.784
**MRD (day 15)**										
<10%	1.00					1.00				
≥10%	0.960	0.381-2.420	0.932	0.001-5.518	0.600	0.859	0.256-2.890	0.807	0.001-88.76	0.334
**WBC count**										
<20000 cells/µl	1.00					1.00				
≥20000 cells/µl	0.823	0.209-3.237	0.781	7.03×10^{-5}-4.191	0.759	0.486	0.106-2.243	0.356	4.85×10^{-6}-2.092	0.240
**BFM risk group**										
Low/Interm. risk	1.00					1.00				
High risk	1.760	0.611-5.069	0.295	0.005-12.27	0.421	0.797	0.244-2.599	0.706	0.002-1.872	0.368
**Age**										
1-5 years	1.00					1.00				
<1 or ≥6 years	2.099	0.701-6.288	0.185	0.693-25.94	0.252	2.439	0.769-7.741	0.130	0.831-22.22	0.103

[a] Hazard Ratio
[b] 95% confidence interval of the estimated HR
[c] calculated by test for trend. Bootstrap *p*-value is based on 1000 bootstrap samples
[d] Bootstrap bias-corrected and accelerated 95% confidence interval of the estimated HR based on 1000 bootstrap samples
[e] Multivariate analysis adjusted for GAS5 expression, WBC count, prednisone response, bone marrow (days 15 & 33), MRD (day 15), risk group and age
